# Effect of OSLM features and gamification motivators on motivation in DGBL: pupils' viewpoint

**DOI:** 10.1186/s40561-022-00195-w

**Published:** 2022-03-16

**Authors:** Angeliki Leonardou, Maria Rigou, Aliki Panagiotarou, John Garofalakis

**Affiliations:** 1 Department of Computer Engineering and Informatics, University of Patras, Rio Campus, 26504 Patras, Greece; 2 Department of Management Science and Technology, University of Patras, Megalou Alexandrou 1, Koukouli, 26334 Patras, Greece

**Keywords:** Digital game-based learning, Interactive learning environments, Media in education, Multiplication game, Open social learner modeling

## Abstract

The primary question of this study is whether OLM and OSLM mechanisms, when used in a digital game, offer higher motivation. Furthermore, the study investigates whether a game’s aesthetics and mechanics support players’ intrinsic motivation. Both claims are tested through the design, implementation and pilot use of the Multiplication Game (MG). MG is a digital learning activity that supports pupils in achieving multiplication competence and provides teacher a dashboard to assess and watch own pupils’ performance. The game enriched with gamification elements to engage and motivate participants. Three versions of the game were used by pupils: without any Open Learner Modeling (OLM) support (i.e. without providing access to own progress data), with OLM support and with Open Social Learner Modeling (OSLM) support, to investigate the difference in motivation among these characteristics. After using the MG for a 2-month period, pupils answered a questionnaire anonymously to express their opinion about MG mechanics, MG aesthetics and intrinsic motivation MG can offer. Furthermore, the corresponding teachers were interviewed to provide insights on their attitude towards MG and its functionalities. A statistically significant difference in Intrinsic Motivation (IM) between the three different MG versions was found and a statistically significant difference in MG Aesthetics and Mechanics between the different grades of primary school. Additionally, Intrinsic Motivation was positively correlated with gamification motivators and MG Aesthetics. Participating teachers stated that MG can improve pupils’ multiplication competence and it is worthy of a stable place in the instructional procedure, as it is a means of pupils’ progress tracking and (self-) assessment, as well as a fun way of practicing and developing multiplication skills.

## Introduction

Designing Web-based learning materials is a quite challenging task as the result should be considered engaging by pupils; its central aim is not to provide information but mainly to facilitate a positive overall pupil experience. As observed by researchers, the Web is sometimes used as an information distribution channel that ignores pupils’ needs (Connolly et al., 2016; Izquierdo-Yusta & Calderon-Monge, 2011; Lopes & Galletta, 2000). Nowadays pupils’ attention cannot be captured merely by the use of the technology; new ways to utilize computers should also be considered in an effort to keep pupils’ attention and motivate them to keep learning in ways that take into account the learning characteristics of each pupil.

Gamification has often been utilized to increase student engagement, motivation and achievement in the classroom with varying degrees of accomplishment (Cahyani, 2016b). Using a gamified learning activity not only can encourage students to try new things, but also can support them overcoming the fear of making mistakes (Cahyani, 2016a; De Moraes Sarmento Rego, 2015; Emel’yanenko et al., 2016; Wood et al., 2013). Moreover, it can lead to a more fun and engaging learning of a topic. Successful and efficient educational applications have a similar relation to instruction, goals, feedback and interaction. Unfortunately, many educators find it difficult to implement in their curriculum strategies to compete with the level of engagement computer games achieve.

Introducing online learning activities with gamification elements in education is a quite promising approach, which can create a new learning culture that appeals to pupils’ habits and interests (Kiili, 2005). The described Multiplication Game (MG) is a digital assessment tool and part of its usefulness resulted from the incorporated logic of digital game-based learning. The game incorporates Open Learner Model (OLM) elements (Leonardou et al., 2019b). An OLM offers to students (and other stakeholders of the learning process) easily perceivable access to personal progress information (Leonardou et al., 2018). Giving students access to view some of their model’s aspects may improve self-reflection, foster self-regulated learning, provide better personalization transparency, and increase user motivation to learn (Al-Shanfari et al., 2017; Bull & Kay, 2013, 2016). Moreover, the MG features a strong social parameter; the social aspect is important in games as social interaction, competition or cooperation can motivate player involvement (Ling et al., 2005; Sweetser & Wyeth, 2005; Yee, 2006a, 2006b). According to the social comparison theory (Festinger, 1954) people have the tendency to compare their achievements and performance with peers', who are considered similar to them with the aim of self-evaluation, self-enhancement and self-improvement, depending on the target of the comparison (i.e. lateral comparison, downward comparison and upward comparison, respectively). As years go by, people become more assured about the general competence of their social comparing skills (Feldman & Ruble, 1977) and a review in the field of social comparison (Dijkstra et al., 2008) concluded that upward comparisons in the classroom often affect pupil performance positively.

The primary question of this study is whether OLM and OSLM mechanisms, when used in a digital game, offer higher motivation by investigating the motivational role of MG in its 3 versions (without OLM features, with OLM features, and with OSLM features). MG is essentially the tool used to study the techniques and mechanisms of OLM, OSLM, and gamification motivators that a digital game should have to enhance motivation. Moreover, it is interesting to examine the potential relationship between intrinsic motivation and MG mechanics (a notion that is equivalent to gamification motivators in the framework of this research), as gamification motivators are known to have a direct relation with extrinsic motivation to game playing (Amir & Ralph, 2014; Dicheva et al., 2014; Kapp, 2012; Muntean, 2011). If a relationship between intrinsic motivation and MG mechanics exists, this could mean that adding gamification motivators in a game is a way to increase players’ intrinsic motivation to keep playing the game. In addition, this study investigates the potential relation between intrinsic motivation and MG aesthetics, which is a correlation that has not been adequately examined in related bibliography (Garcìa-Vergara et al., 2015)(Alexiou & Schippers, 2018). A relationship between these two factors would practically mean that when a game incorporates aesthetic elements such as sound, graphics and animation, and also makes users feel enjoyment, satisfaction, pleasure, envy, respect, connection as suggested by the MDA framework (Amir et al., 2015; Hunicke et al., 2004), then players become intrinsically motivated to play it. To this end, a survey has been conducted addressing elementary school pupils who played MG under the supervision of their teachers for a period of two months and answered anonymously the questionnaire that was designed for the purposes of this research. As motivation, MG aesthetics and mechanics are not directly observable, confirmatory factor analysis is employed to estimate and test measurement models incorporating indicator variables for these latent constructs.

## Related work

### Motivation

Motivation is considered to be an urge that leads to changes in behavior and particular actions (Brown, 2007; *Incentive Theory of Motivation and Intrinsic vs. Extrinsic Motivation*, n.d.; Weiner, 1990). Its role in learning and achievement in everyday life is very important in both formal and informal learning scenarios, and as pointed out in (Pintrich, 2003) motivated pupils are more engaged, persist longer, have better learning outcomes, and perform better in comparison to non-motivated peers on standardized achievement tests. There are 3 basic theories to explain different aspects of motivation (Bandura, 1997; Hodges, 2004): *attribution theory*, *expectancy-value theory* and *goal theory and are all c*losely connected to the concept of motivators. Motivators provide some sort of incentive for completing a task. There are two categories of motivation based on the nature of the motivator (Hodges, 2004): intrinsic deriving from internal factors and extrinsic deriving from external factors (*Incentive Theory of Motivation and Intrinsic vs. Extrinsic Motivation.*, n.d.).

Intrinsic motivation refers to learner’s internal desire to perform an activity and reach reward like personal satisfaction, enjoyment and feelings of competence and self-determination (Deci, 1975; Hodges, 2004). Intrinsic motivators can be passion with the topic, level of relevance with everyday life and its context, sense of achieving competence. Intrinsic motivation can be long-lasting and self-sustaining, therefore behavior will be influenced in a slow manner, while a personalized and time-consuming preparation is necessary. Due to the diversity of learners, different approaches should be utilized to motivate everyone (DeLong & Winter, 2002). Based on the nature of the internalized utility of the behavior, intrinsic motivation can have 3 forms. (Vallerand et al., 1992):

- *Intrinsic motivation to know*, when a learner experiences the desire to perform a learning activity for the pleasure one experiences while learning (i.e. the utility to an individual is the learning in and of itself).
- *Intrinsic motivation towards accomplishment,* when a learner desires to engage in an activity for the pleasure and satisfaction experienced when accomplishing a difficult feat.
- *Intrinsic motivation to experience stimulation* occurs when a person takes part in an activity in order to be stimulated. Stimulation can take a range of forms such as sensory pleasure, aesthetic pleasure or emotional sensations such as fear or excitement. (Buckley & Doyle, 2016)

Extrinsic motivation appears when a learner is engaged in the activity not for the subject or the content but because it is a necessary path leading to target (Harlen & Deakin Crick, 2003). In case that a learner is motivated by rewards and incentives external to personal interest and satisfaction, then these factors are extrinsic motivators (Hodges, 2004) which can be money, prize, grades, positive feedback (Brown, 2007), or the learner’s purpose to satisfy parents, the desire to attain high assessment in an external exam and to be best among peers (DeLong & Winter, 2002; Ur, 1996). According to Deci (Deci et al., 2001), extrinsic motivation can also has also been refined into more precise constructs (Deci et al., 2001). Although the stimulation prompting behavior is always external to the participant, extrinsic motivation can take 3 forms due to each one possessing a different grade of participant’s autonomy:

- *External regulation* is the least autonomous form of extrinsic motivation. The participant’s behavior is shaped to achieve satisfaction of an external demand, to meet an externally set standard or to avoid an externally imposed penalty. Typically, these behaviors are externally imposed.
- *Introjected regulation,* a second form of extrinsic regulation, describes the situation when activities are driven either by self-esteem strengthening or by the urge to avoid feeling guilty. Although the regulation is internal to the participant, the stimulus is external.
- *Regulation through identification* is the most autonomous form of extrinsic motivation. In this form a bond is observed between participant’s identity and an externally proscribed behavior and therefore (s)he behaves in order that own identity to be supported (Buckley & Doyle, 2016).

In this study as already mentioned, the focus is on intrinsic motivation and its relation with game mechanics and aesthetics.

### Motivation and digital games

*Digital game-based learning* (DGBL) derived from the union of interactive entertainment and serious learning through digital games (Prensky, 2001). Therefore, DGBL contains two parameters: learning (education) and gaming (fun, entertainment) (Bellotti et al., 2013; Nussbaum & Beserra, 2014). The entertaining perspective of digital games in order to support specific educational purposes had the initial aim of promoting motivation. “*Motivation is a condition that activates and sustains behavior towards a goal*” (National Academies of Sciences Engineering and Medicine, 2018) (p. 109). Motivation plays a central role in learning and achievement on many levels of everyone’s life, as well as in both formal and informal learning scenarios. If pupils are motivated, then they are engaged, persist longer, have better learning outcomes, and perform better in comparison with non-motivated peers on standardized achievement tests (Pintrich, 2003). According to the definition given by Brown, motivation is “*an inner drive, impulse, emotion, or desire that moves one to a particular action*” (Brown, 2007) (p.114). Therefore, a motivated learner is the learner “*who wants to achieve a goal and who is willing to invest time and effort in reaching that goal*” (Daskalovska et al., 2012) (p.1187). As intrinsic motivation (see paragraph 3.7 for details) is characterized by a learner’s internal desire to perform a task and can only be rewarded with personal satisfaction and enjoyment, it thus derives from the learners and their attitudes toward the topic, their learning goals and aims, their emotions, and their ambitions (Daskalovska et al., 2012; Hodges, 2004; Leonardou et al., 2020). Intrinsic motivation along with learning deriving from fun, autonomy and experiential learning are defined as the main concepts constructing DGBL (Perrotta et al., 2013).

The nature of games can support learners’ engagement and involvement, motivation and interest, and at the same time the retention of learned skills (Cahyani, 2016b). Game-like elements can be used in educational settings, for example in the case of deploying avatars players may gain social credibility and recognition. Furthermore, good game designs perfectly match the player’s cognitive abilities with the difficulty level and also games give learners the opportunity to learn from mistakes in quick recovery (Lee & Hammer, 2011). According to Cahyani (2016a, 2016b) “*Gamification within learning process allows students to fail and not feel rejected, so they are willing to try more and more*” (p.3). Despite the fact that games’ central aim is entertainment, they also support a plethora of other aspects like training and knowledge sharing in domains such as defense, education, scientific exploration, healthcare, emergency management, city planning, engineering, religion, government and non-governmental organizations (NGOs), business, marketing, communication and politics (Breuer & Bente, 2010; Muntean, 2011; Susi & Johannesson, 2007). In coherence to serious games (games targeting at investigating, training, and advertising Breuer & Bente, 2010; Muntean, 2011; Susi & Johannesson, 2007)), gamification is the application of game elements for purposes that go beyond mere entertainment (Deterding et al., 2011a, Deterding et al., 2011b). Both serious games and gamification try to reclaim games’ characteristics with the aim to achieve something beyond playfulness. Gamification utilizes game-based mechanics, aesthetics and game thinking in order to promote engagement, motivation, to support learning and solve problems (Kapp, 2012). Gamification is the adoption of game-design elements and game rules in non-game contexts in order to improve user experience, motivation and engagement, specifically in non-game contexts (Groh, 2012). It can also be defined as an online interactive system design that makes use of people's desire for competitive and rewards to motivate the player (Anderson & Rainie, 2012). Rewards can be virtual rewards e.g. payments, points, badges, free gifts (Cahyani, 2016b). One term used to identify different types of rewards is SAPS—Status, Access, Power and Stuff (Zichermann & Cunningham, 2011). The reward can often indicate the level of competence that has been achieved. Reward systems also use progress tracking (Buckley & Doyle, 2016).

The gamification element is based on the MDA framework (Hunicke et al., 2004). According to the MDA Framework, a game needs to possess 3 aspects: (a) Mechanics, describing the specific parts of the game, at the data representation level and algorithms, (b) Dynamics, describing the describes the behavior of the mechanics acting—during game- on player inputs and each others’ outputs, and (c) Aesthetics, describing the desirable emotional responses of the player while interacting with the game. According to Amir et al. (2015) game mechanics can be points, levels, challenges, virtual goods, leaderboards, badges, gifts and charity; game dynamics can be reward, status, achievement, self-expression, competition, altruism, and aesthetics can be satisfaction, pleasure, envy, respect, connection. On the other hand, Hunicke et al. (2004) supported that the aesthetics of a game comprise: sensation (game as sense-pleasure), fantasy (game as make-believe), narrative (game as drama), challenge (game as obstacle course), fellowship (game as social framework), discovery (game as uncharted territory), expression (game as self-discovery), and submission (game as pastime).

Among the typical game design elements, those with the strongest effect on motivation are points, badges, leaderboards, performance graphs, meaningful stories, avatars and teammates (Sailer et al., 2017) that also are analytically presented in (Leonardou et al., 2020). All these game design elements share strong motivational influence as supported by the self-determination theory (Deci & Ryan, 1985; Ryan & Deci, 2000). According to this theory, behavior is strongly determined by three universal, innate, psychological needs: autonomy, competence and social relatedness. In an effort to correlate these three intrinsic psychological needs with the game-based elements, it is easy to observe that as collected points are immediately influenced by player’s actions, they offer a quantified view of player’s progress and therefore the need for competence is addressed (Sailer et al., 2017). Performance graphs represent not only player’s performance, but also competencies’ level and thus the need for competence is satisfied. The need for competence is also met in badges, as badges are directly connected to player’s progress and in leaderboards, as they visually rank players’ performance. As the need for autonomy can be expressed in two forms: experience of decision freedom and experience of task meaningfulness, it can be claimed that avatars satisfy this need due to the freedom of choice they offer to players (Peng et al., 2012). On the other hand, meaningful stories satisfy the second aspect of the need for autonomy, as through stories players experience their choices meaningfully and in an engaging manner (Rigby & Ryan, 2011; Sailer et al., 2017). On one hand, gamification motivators as clearly extrinsic and independent elements to learning, are obvious part of the extrinsic motivation a game can offer to the user (B. Amir & Ralph, 2014; Dicheva et al., 2014; Kapp, 2012; Muntean, 2011). On the other hand, due to the bond gamification motivators possess with autonomy, competence and social relatedness, which are intrinsic psychological needs, it is more recently claimed that they are positively related to intrinsic motivation, as well (Matallaoui et al., 2017; Richter et al., 2015a, 2015b; Yang et al., 2020). This latter conclusion is reached through this study as well.

## Game description

As fluency in the multiplication table is considered a very significant ability, it is crucial to support pupils with modern educational tools (Caron, 2007; Gersten & Chard, 1999) rather than use only traditional ways of learning and practicing. There are many software applications in the related bibliography with the aim not only to support the teachers’ role but also to motivate pupils to attend and participate in plethora of lessons. Several games and educational applications have been developed for pupils to practice multiplication facts in an easier and more enjoyable way; nevertheless, the memorization of these facts can be a laborious and prolonged task for pupils in primary education (Baroody, 1985; Caron, 2007; Davis, 1984; Gagné, 2018; Gersten & Chard, 1999; National Council of Teachers of Mathematics, 2000). In addition, specific multiplication facts are proved to be even more difficult to memorize by both children and adults and therefore need more time to learn (van der Ven et al., 2015).

The gameplay is the central part of every game therefore its importance should be taken into consideration, as a good gameplay keeps a player motivated and engaged throughout the entire game (Costkyan, 2002). Gameplay can be defined as “*the interactive gaming process of the player with the game*” (p.1) (Nacke et al., 2009) and moreover “*the experience of gameplay is one of interacting with a game design in the performance of cognitive tasks, with a variety of emotions arising from or associated with different elements of motivation, task performance and completion*” (p.1) (Craig et al., 2008). The gameplay also includes all players’ actions that should be executed to deal with challenges. In educational game design educational goals on one hand and gameplay on the other, should reach a balance, so as to achieve a meaningful entity (Kiili, 2005). Moreover, and since the MG addresses schoolchildren, it is crucial to deploy pleasant graphics, bright colors, related sound effects, and animation so that it visually allures learners and thus engages them.

Flow experience is also a central notion and it is closely related to gameplay. According to Csikszentmihalyi (2008), flow experience is a situation where people enjoy and concentrate on an activity. Researchers (Ghani & Deshpande, 1994) supported that immersion during an activity without external interruptions, produces concentration and enjoyment. Studies (Kiili, 2005) concerning learners' flow experiences toward games indicated that learners both focused on learning and were active in a game-based environment, furthermore it was pointed out that simulation games support university students' flow experiences, especially for a sense of control, clear goals, challenge-skill balances, rewarding experiences, and feedback. These experiences lead to learners’ feelings of pleasure and exhilaration. When challenging tasks in game-based learning are too simple, people can easily get bored, while when they are too difficult, people can feel frustration and disappointment (Chang et al., 2017). If difficulty levels in a challenging task are compatible to learners' abilities, then they can feel pleased and joyful (Csikszentmihalyi, 2008). Another study evaluating learners' learning achievements and flow experiences (Admiraal et al., 2011) proved that learners had higher flow experiences in games, and furthermore had a better understanding of the learning contents. MG comprises flow experience’s main constructs as clear goals are set in each level*,* feedback is offered after user’s answer, sense of control is provided through personalization graphical elements (avatars, user’s name) and rewarding experience is reached through coins’ gaining. Additionally, MG’s difficulty can range and be tailored to the instructional procedure, as the player can select to perform only multiplication facts already been taught.

**Fig. 1:**
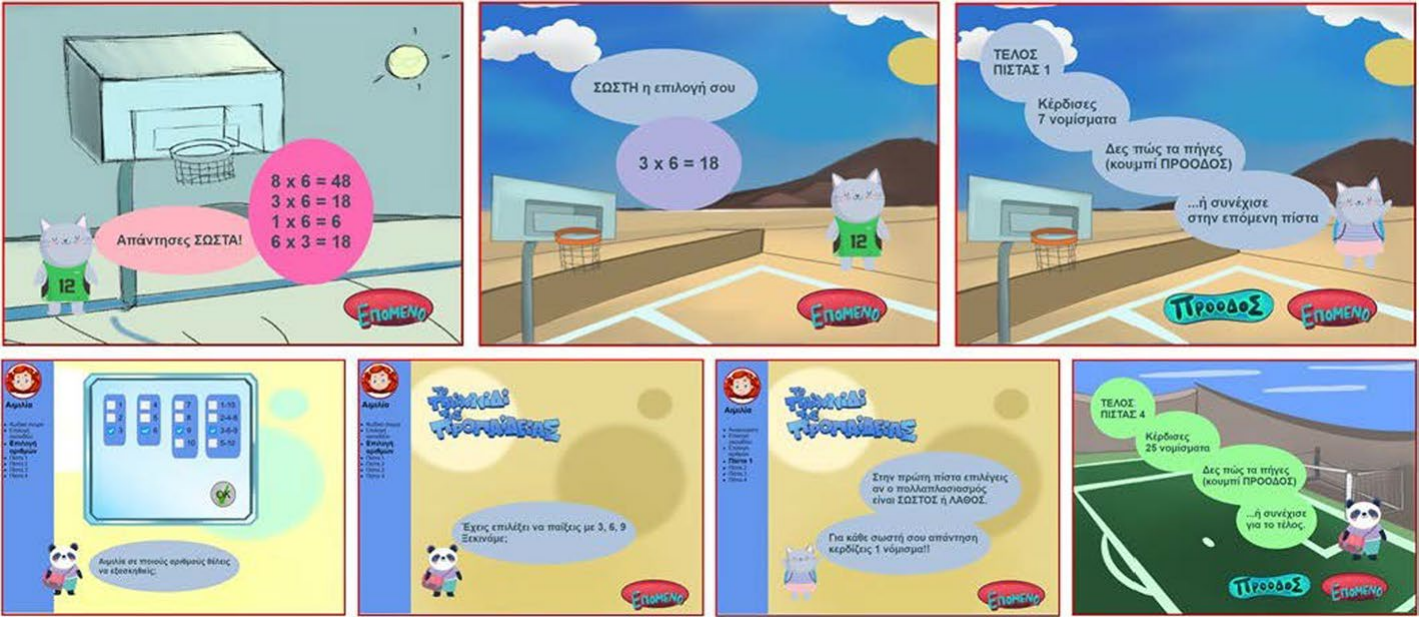
NPC characters

MG extends the educational game idea with an adaptation mechanism, the notion of OLM and the introduction of a social parameter. It aspires to support pupils in learning multiplication table in a way that engages and motivates them and furthermore to investigate the effect of allowing access to the progress of peers and summative class scores. It is a web-based practice and progress monitoring application supporting scientific and educational aims. More details on how MG functions are presented in previous studies (Leonardou et al., 2019a, 2019b; Leonardou et al., 2021a, 2021b). This study focuses on the incorporation of gamification motivators in this last MG version aimed at enhancing MG’s motivational role: points (coins), avatar icon, visualization of level achievements (current and previous), NPC characters giving information (Fig. 1), children-friendly graphics and sound effects, social comparison and leaderboards. Studies (Brusilovsky et al., 2016; Hsiao & Brusilovsky, 2012; Hsiao et al., 2013) pointed out that through accessing peers’ models, students cover more topics in the system and reach higher success rates in self-assessment problems.

**Fig. 2:**
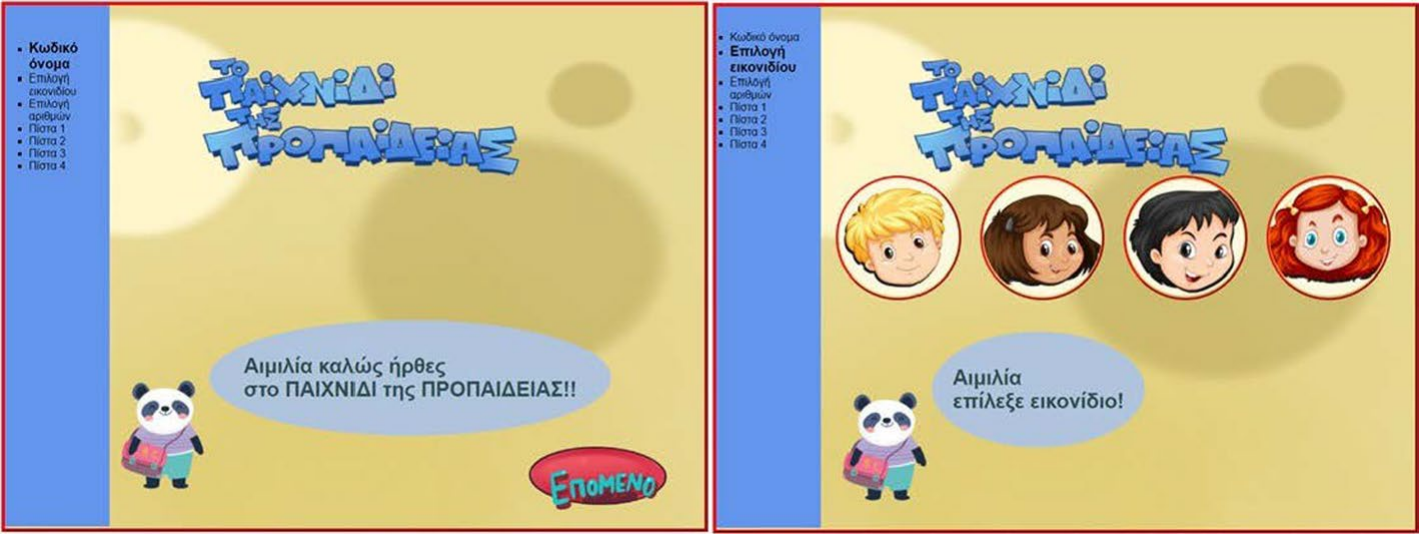
Personalization features in MG

**Fig. 3:**
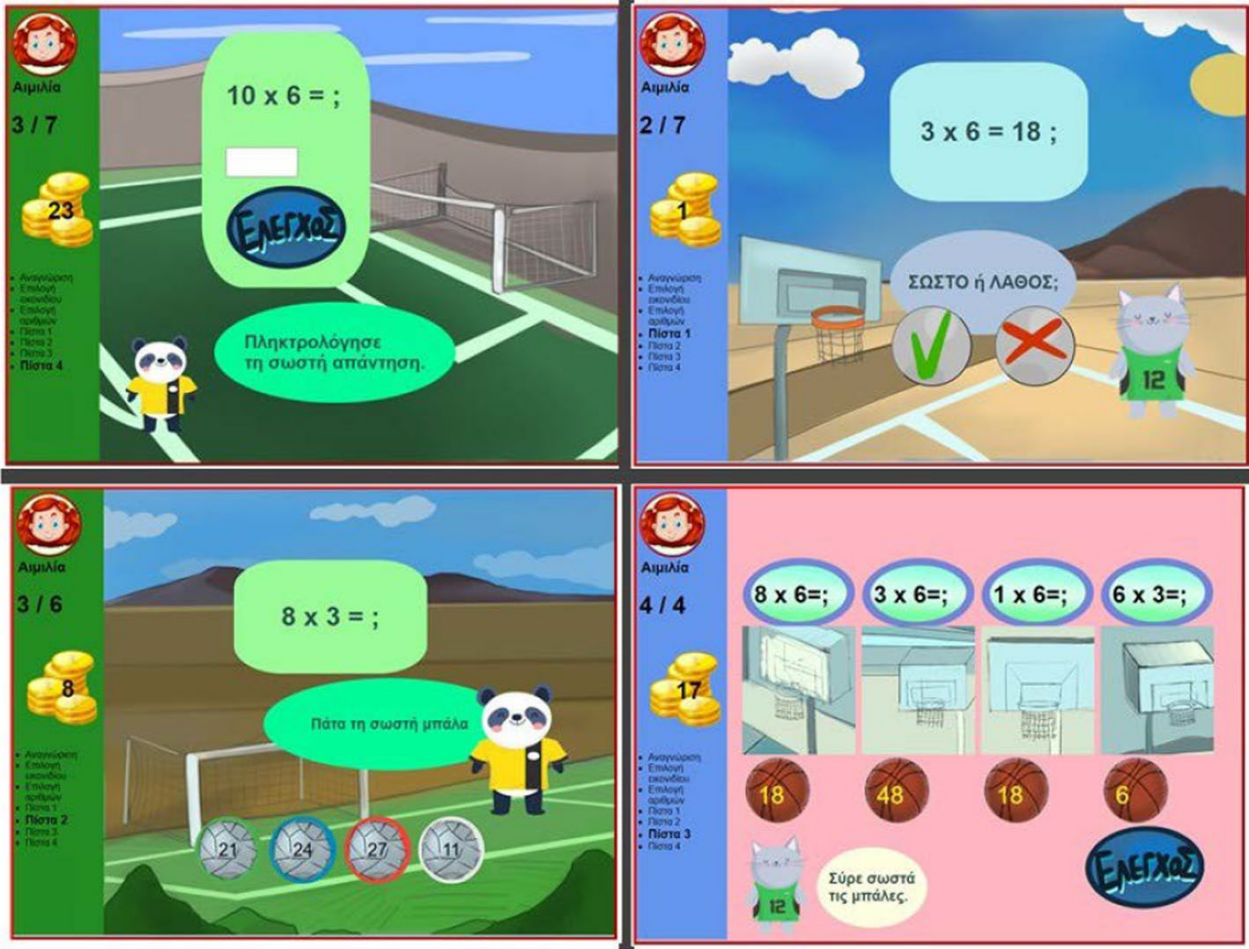
MG’s levels

**Fig. 4:**
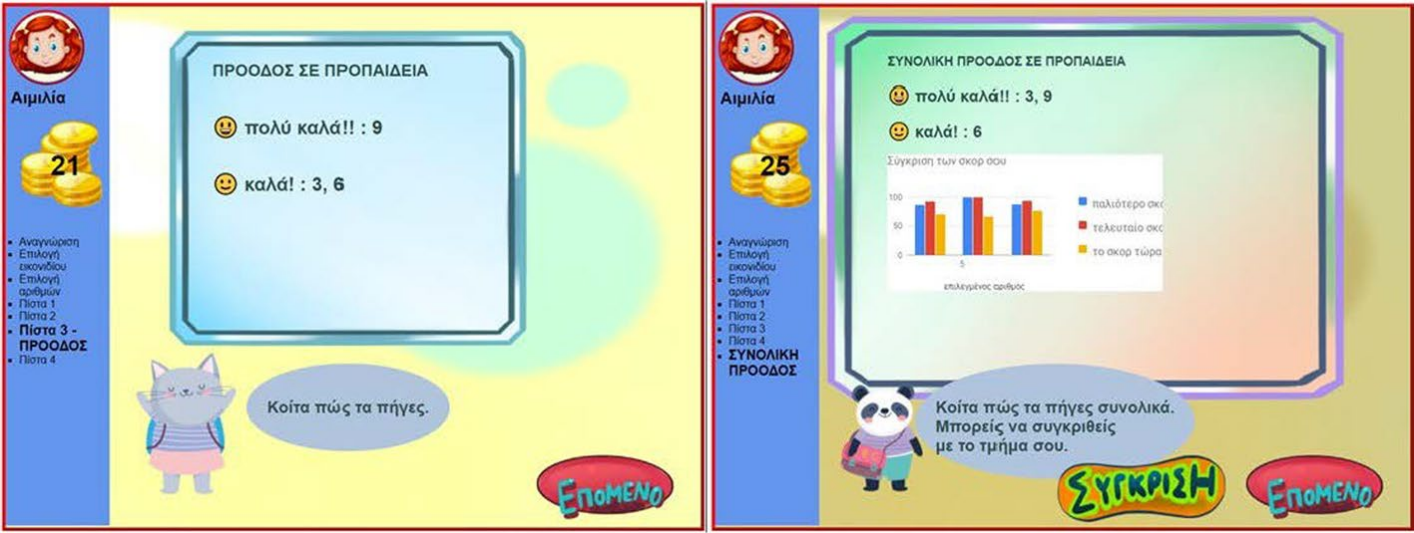
Performance graphs in MG

**Fig. 5:**
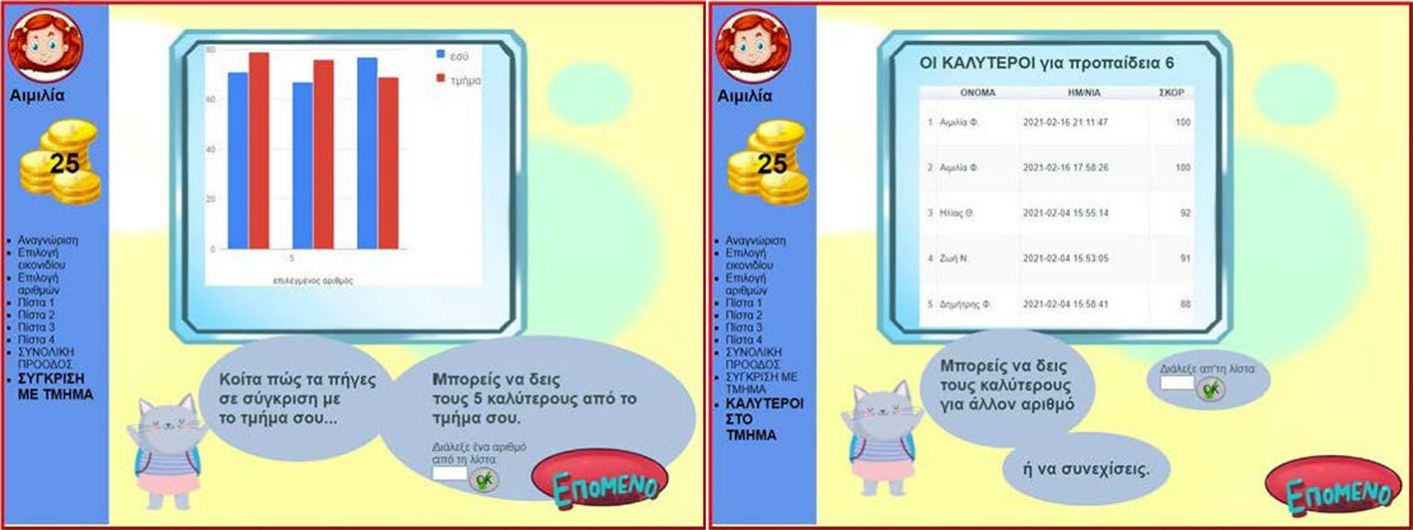
Social opening of LM in MG

As MG is a personalized activity, when the pupil is uniquely identified by a nickname and can chose among four avatars (Fig. 2). The game consists of four levels (Fig. 3). Upon completion of each level in MG, the player has two choices: either to continue to the next level, or to see the progress achieved so far in visual and textual form (Fig. 4). Information is visualized as smiley faces (i.e. simple quantized representations suitable for primary school ages (Bull & McKay, 2004)). Another choice offered is the comparison of the specific player to the classroom’s aggregated progress. Additionally, a Hall of Fame is presented for each of the selected numbers, with the names of the classmates holding the best corresponding scores ordered by recentness in case of equal scores (Fig. 5).

Through the top five ranking pupils, recognition is achieved for high-scoring players, whereas low-scoring players are in no way ‘exposed’. On the other hand, as every pupil belongs to a certain group (own classroom), the notion of teammates is dominant since all the pupils appearing in the Hall of Fame belong to the same classroom (a pupil only sees his classmates). According to Richter et al. (Ganit Richter et al., 2015a, 2015b), “*a combination of a progress bar and a leaderboard is likely to generate excitement, commitment, a will to finish a gamified activity in a successful manner, and even desire to repeat the experience*” (p.38). From another point of view, as these choices are optional (access to the level or game achievements, access to the HALL of Fame or to class average scores, etc.), flow experience is not disturbed, as the user isn’t interrupted while playing, but is given the option to access this information when moving from one level to the next.

**Fig. 6:**
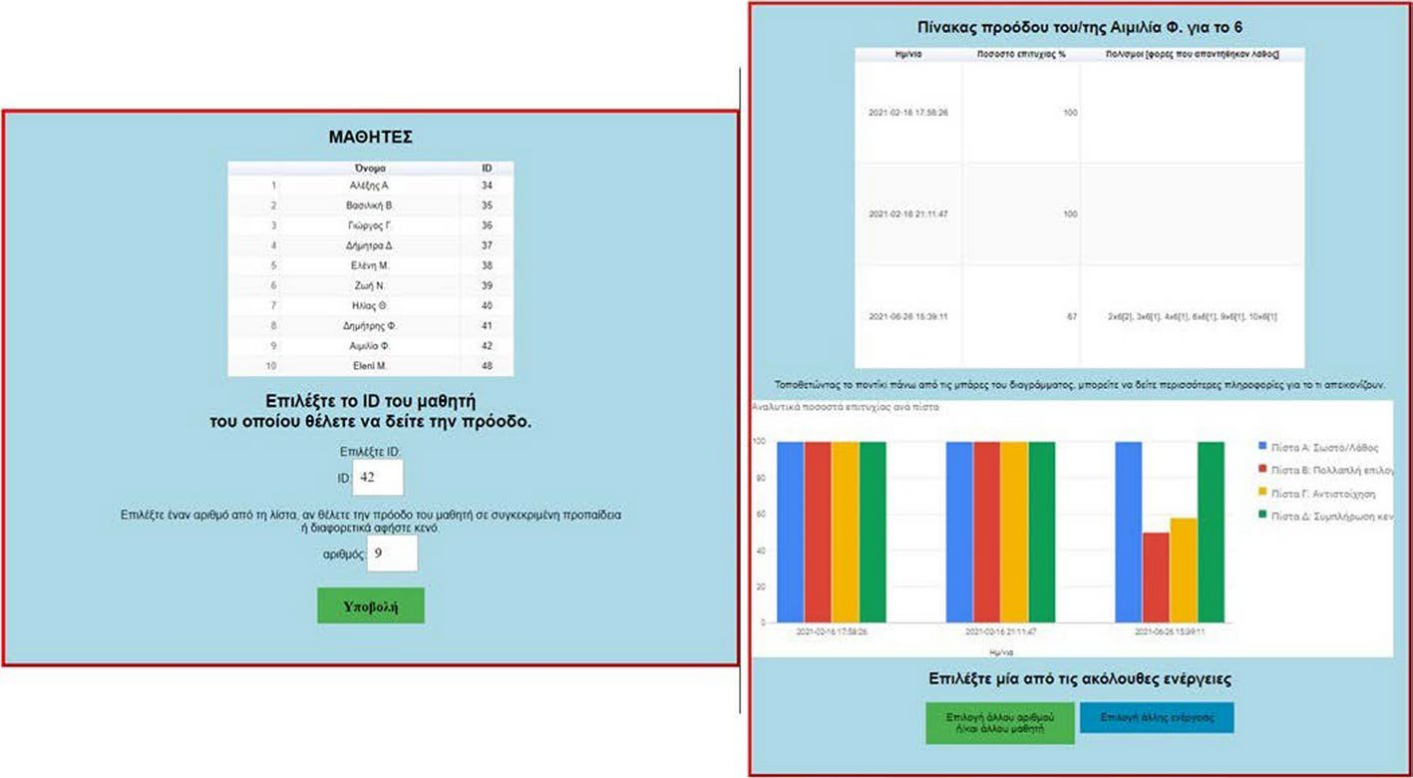
Social opening of MG (teacher aspect 1)

**Fig. 7:**
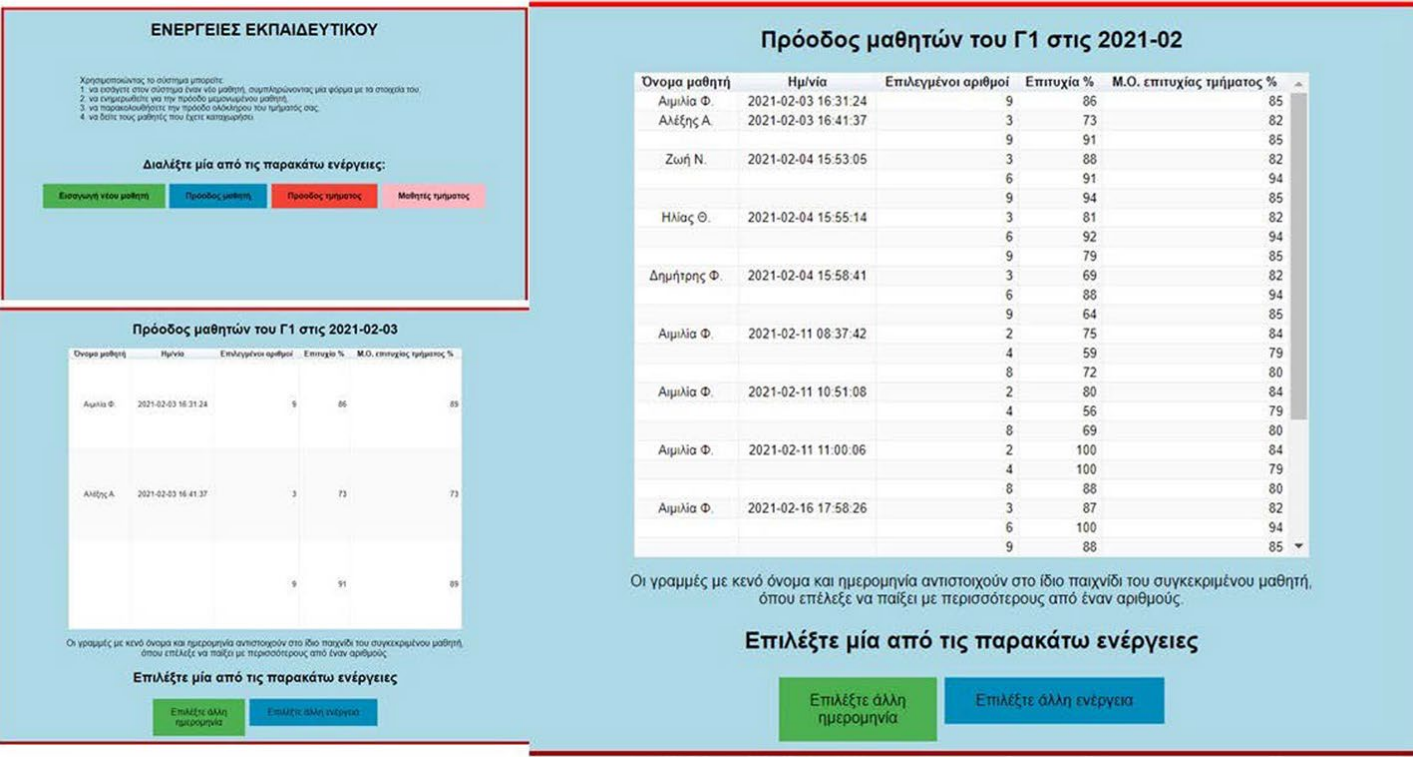
Social opening of MG (teacher aspect 2)

Another significant feature of MG concerns the teacher: (1) visualization of the progress of individual pupils belonging to the teacher’s class (Fig. 6), and (2) visualization of the aggregated progress of all pupils in the class (Fig. 7), for more details see (Leonardou et al., 2021a, 2021b).

Considering the main learning theories, the Multiplication Game is firstly influenced by the behaviorist learning theory (Padgett, 2020; Skinner, 2009; Watson, 2013), as in behaviorism’s context the practices that are used in the classroom are practice and repetition in a more active way, and so in MG displayed possible answers to multiplication facts are intentionally selected to be informative distracters (Buwalda et al., 2016); therefore, a correct answer indicates genuine learning rather a lucky guess. On the other hand, MG offers rewards through gamification motivators (e.g. coins). The game also adapts the Cognitive Learning Theory’s approach (Cooper, 1993; Ertmer & Newby, 2013; Piaget, 1976), as the learner is expected to be active, while (s)he receives information during the first scenes of the game, as in both cases of right and wrong answers, the correctly completed multiplication is given and finally in the last scene user is expected to utilize that information to produce learning outcomes (fill in the gap questions). Furthermore, MG uses social interaction to support learning as Constructive Theory indicates (Cooper, 1993; Ertmer & Newby, 2013; Piaget, 2013; Vygotsky, 1978). Also, groups of numbers—that a user can choose to exercise on the corresponding multiplication tables in the MG—are constructed in a way that the knowledge of multiplication tables of the latter members to be built based on the knowledge of the first members (2–4-8, 3–6-9, 5–10). It can be supported that MG’s adaptiveness is in correspondence to the opinion expressed in Humanist Learning Theory (DeCarvalho, 1991; Huitt, 2009; Rogers & Freiberg, 1994) that personal needs of every user should be identified in order (s)he to be supported. Finally, MG is a digital learning tool that also socially opens LM to peers and teacher and thus leads to a connected community – as Connectivism suggests (Downes, 2010; Siemens, 2004)—for the learning purpose of multiplication tables.

## Research design

### Scope

The primary purpose of this study was to investigate the effect (if any) of Open Learner Modeling and Open Social Learner Modeling features on motivation. This survey was conducted to investigate whether gender, age and MG version influence pupils’ opinion on intrinsic motivation that MG offers and on MG mechanics and aesthetics. Furthermore, the study focuses on gamification motivators of MG and their possible relation with motivation triggered by MG. For this reason, three constructs were formed and confirmed by the Structural Equation Model (SEM) and checked the following research questions:

- Are there significant differences in pupils’ intrinsic motivation (IM) for using MG among the different MG versions?
- Are there significant differences in pupils’ attitudes to MG mechanics (MGM) and MG aesthetics (MGA) among the different MG versions?
- What are the relationships between pupils’ attitudes to MG mechanics (MGM) and their intrinsic motivation (IM)?
- What are the relationships between pupils’ attitudes to MG aesthetics (MGA) and their intrinsic motivation (IM)?
- Are there significant differences in pupils’ intrinsic motivation (IM) between female and male students and across the different grades of the pupils (pupil age)?

Therefore, five hypotheses were set based on questions (a) to (e):

- H1: MG mechanics (MGM) is positively related to intrinsic motivation (IM).
- H2: MG aesthetics (MGA) is positively related to intrinsic motivation (IM).
- H3: Intrinsic motivation (IM), MG aesthetics (MGA) and MG mechanics (MGM) are different across different MG versions
- H4: Intrinsic motivation (IM), MG aesthetics (MGA) and MG mechanics (MGM) are different across different pupil grades
- H5: Intrinsic motivation (IM), MG aesthetics (MGA) and MG mechanics (MGM) are different between male and female pupils

### Participants

For the purpose of the present study, 137 pupils of 1st to 3rd grade of Greek public primary schools were involved (Table 1). The game is primarily an assessment tool that supports learning through the feedback pupils receive after correct and wrong answers. So, it is assumed that the basic learning process takes place in the traditional classroom, the traditional way and the game is introduced in this framework as a supportive mechanism. Pupils are registered in the game by their schoolteacher and the teacher suggests how and when to use the game and oversees pupil activity. Thus, the role of the school itself and the schoolteacher remain central. Before the study, the teachers were informed about MG and its OLM/ OSLM characteristics through a video. Next, they presented MG to their pupils in distance learning conditions due to covid-19 pandemic. No extra support was provided before playing MG for first time, as the gameplay is simple and clear. Indeed, as it turned out even 1st grade pupils did not encounter any problems in using the game.

**Table 1:** Demographic profile of 137 respondents

	Frequency	Percentage (%)
Gender		
Boy	72	52.6
Girl	65	47.4
MG version		
Version 1 (without any OLM element)	29	21.2
Version 2 (with OLM elements)	49	35.8
Version 3 (with OSLM elements)	59	43.1
Grade		
1st	17	12.4
2nd	77	56.2
3rd	43	31.4
Times played MG		
Less than 5	99	72.3
5–10	29	21.2
More than 10	9	6.6
Amount of numbers selected		
Less than 5	52	38.0
5–15	42	30.7
More than 15	43	31.4

Prior to the beginning of the pilot study, students’ parents were briefed, and their written consent for their children’s participation was obtained. Furthermore, parents were informed about the fact that pupils can also play MG at their own time. Additionally, they were advised not to urge their child to play the game, but only to give their child the opportunity to do so, for example by helping them connect online or intervene in case of technical issues. Additionally, each teacher created an account for every pupil.

Three versions of MG were used: one without OLM characteristics and 2 with OLM and OSLM characteristics respectively. Although the formal curriculum of Greek public schools suggests the teaching of multiplication tables (numbers 1, 2,5, 10) in the 1st grade, in many cases teachers choose not to teach them, due to lack of time. This explains the low percentage of 1st grade participants in this research.

### Measurement

A short questionnaire was implemented and was distributed to the groups of students who used the game to record their views and attitudes towards MG characteristics (mechanism, aesthetics) and its motivation role. The questionnaire was composed of 4 sections comprising a type of 5-point Likert scale questions called smileyometer (ranging from 1 which means ‘‘strongly unhappy’’ to 5 which means ‘‘strongly happy’’) (Gena et al., 2020; Read et al., 2002; Read & MacFarlane, 2006; Sim & Horton, 2012; Zaman et al., 2013). The first section records the demographics of participants and details of their playing including gender, grade, times of playing MG, count of numbers selected in total. The second section refers to the motivational role of MG and more specifically to intrinsic motivation (IM), the third refers to MG mechanics (MGM) which at the same time depicts the Gamification Motivators (GM) factor based on the selection of the questions, and the fourth to MG aesthetics (MGA) (Table 2). The assessment tool contained 3 factors and 12 Questions. Within them, factors represented by questions were created, aiming to assess the pupils’ belief about MG characteristics. Each subscale comprised 3–6 items.

**Table 2:** Factors and Questionnaire items

Factor	Questionnaire Item (see appendix for the pupil questionnaire)	Cronbach’s alpha
Intrinsic Motivation (IM)	IM1, IM2, IM3	0.776
MG Aesthetics (MGA)	MGA1, MGA2, MGA3	0.726
MG Mechanics/ Gamification Motivators (MGM/GM)	MGM1, MGM2, MGM3, MGM4, MGM5, MGM6	**0.688**

Bold is used to emphasize that factor MGM/GM has a cronbach value below the limit of 0.7

### Results

#### Demographics

Table 1 describes the demographic characteristics of the participants. As seen in Table 1, 65 (47.4%) of the participants were female and 72 (52.6%) were male. Of the participants, 29 (21.2%) played the MG without OLM, 49 (35.8%) played with OLM characteristics, and 59 (43.1%) with OLM and OSLM characteristics. Pupils were grouped according to three grades (1st to 3rd) and pupils randomly sampled in the selected classes (17 (12.4%) for 1st grade, 77 (56.2%) for 2nd grade and 43 (31.4%) for 3rd grade) and filled in the questionnaires. As concerns the times that pupils played the MG, the majority of participants played less than 5 times (99 (72.3%)), while 52 pupils (38%) selected less than 5 numbers in total in their playing, 42 (30.7%) selected from 5 to 15 number in total and 43 pupils (31.4%) selected in total more than 15 numbers (note that what is counted by “amount of numbers selected” is the total selected numbers to practice in all pupil game sessions and not their unique occurrences).

#### Instrument design

In this study three components are used to understand the pupils’ attitude towards use of MG, measuring the following aspects as shown in Table 2: (1) Intrinsic Motivation, (2) MG Aesthetics, and (3) Gamification Motivators.

In the analysis, the reliability of the questionnaire was assessed using Cronbach’s Alpha Model, a model of internal consistency. The values that are in the accepted level of reliability (Abe & Gbenro, 2014) prove that the factors are quite reliable for data collection, and the reliability coefficients of the items used was calculated above 0,7 (except GM that was calculated nearby as 0.7) indicating that the reliability coefficient of the data collection instrument is.

## Data analysis

Structural Equation Model is used to analyze the structural relationship between measured variables and the three latent constructs, as well to apply multiple regression analysis (Tarka, 2017). The validity and internal reliability were tested using AMOS, an extension of SPSS statistical software. KMO and Bartlett’s test were used to determine the feasibility of the component analysis and, convergent validity was evaluated by examining composite reliability (CR), and average variance reliability (AVE). Several goodness-of-fit measures have been checked, after fitting the proposed model. In testing different combinations of variables, three constructs were established, and 12 variables were included: IM with 3 items (IM1, IM2, IM3), MGM with 3 items (MGM1, MGM2, MGM3), MGA with 6 items (MGA1, MGA2, MGA3, MGA4, MGA5, MGA6). The means and standard deviations for three factors and corresponding items are shown in Table 3. The mean scores of factors range from 4.34 to 4.55 and demonstrate pupils’ positive attitude towards IM, MGM and MGA.

**Table 3:** Descriptive statistics for factors

	Mean	SD
IM	**4.34**	**0.42**
IM1	3.91	0.452
IM2	4.55	0.555
IM3	4.58	0.537
MGM	**4.48**	**0.43**
MGM1	4.44	0.651
MGM2	4.55	0.568
MGM3	4.49	0.654
MGM4	4.47	0.687
MGM5	4.61	0.559
MGM6	4.36	0.838
MGA	**4.55**	**0.45**
MGA1	4.52	0.608
MGA2	4.48	0.654
MGA3	4.66	0.474

Bold is used to distinguish the three factors (IM, MGM, MGA)

KMO and Bartlett’s value was 0.790 (*p* < 0.0001) indicating the suitability of the sample size. Principal component analysis was thus employed to examine the factor validity. Three factors were extracted and were consistent with the hypothesized construct and all eigenvalues were not greater than 1 according to the Kaiser criterion. The model explains 58.9% of the total variance so the questionnaire holds construct validity (according to the data).

Indicators of the model factors’ reliability and validity were calculated (Table 4). The reliability of each factor exceeded the 0.7 threshold except for MGA that is considered moderate, but acceptable (Taber, 2017). Therefore, the measurement scales were valid and reliable and composite reliability and convergent validity was achieved for the three factors.

**Table 4:** Reliability statistics

Factor	Items	CR	AVE
IM	3	0.74	0.5
MGM	6	0.85	0.4
MGA	3	0.7	0.5

**Fig. 8:**
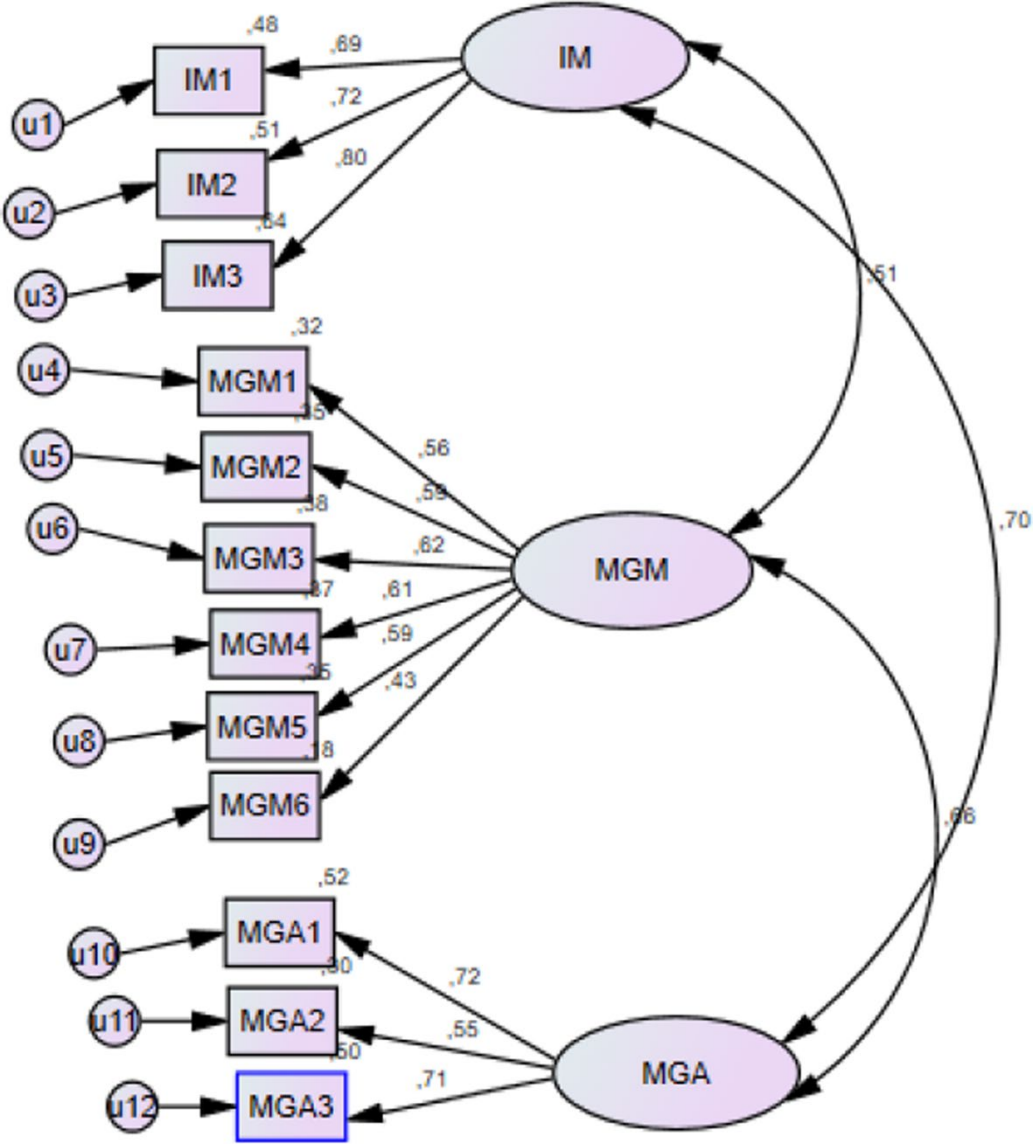
Structural Equation Model

Based on the proposed research model and data analysis discussed in the previous section, the structural model was confirmed (Fig. 8). All specified paths between the model factors had significant coefficients. The strongest effect was recorded with IM on MGA (0.70), and the weakest with IM on MGM (0.51).

The fit indices show good fitness of the model with the sample data which means that the structural model fits the data satisfactorily (Table 5).

**Table 5:** Indicators for measuring the model’s adequacy

Indicator	Expected value	Value
CMIN		94,484
df		51
RMSEA	< 0.05	0.054
RMR	< 0.05	0.023
NFI	> 0.90	0.811
CFI	> 0.90	0.9
GFI	> 0.906	0.906

### Differences between MG version, grade and gender

To examine possible relation (hypothesis tests H2–H4) between factors and different group nonparametric tests were conducted due to the fact that data are not normally distributed. First as concerns the H2, different MG versions used and the factors, Kruskal Wallis test is selected, which is a non-parametric test that checks as a null hypothesis if the groups have equal mean means (random order), i.e. that a group differentiates the data order. For the application of the method the data are arranged in ascending order and in each data its relative position is determined which is called mean rank (Table 6).

**Table 6:** Test statistics [Kruskal Wallis Test—Grouping Variable: MG version]

Ranks			
	MG version	N	Mean rank
IM	Version 1 (without any OLM element)	21	11.81
	Version 2 (with OLM elements)	69	100.58
	Version 3 (with OSLM elements)	47	48.19
	Total	137	
MGM	Version 1 (without any OLM element)	21	68.84
	Version 2 (with OLM elements)	69	85.40
	Version 3 (with OSLM elements)	47	55.46
	Total	137	
MGA	Version 1 (without any OLM element)	21	31.64
	Version 2 (with OLM elements)	69	84.59
	Version 3 (with OSLM elements)	47	62.80
	Total	137	

It was demonstrated that there is effect from different MG versions in MG Intrinsic Motivation (H(2) = 111,790, *p* < .001),MG Mechanics (H(2) = 10,938, *p* = 0,04 < .05) and in MG Aesthetics (H(2) = 33,069, *p* < .001).

As seen in Table 6, Version 2 leads to higher IM, MGA and MGM.

In order to examine possible relation between different ages (grades) of participants and the factors, Kruskal Wallis test was conducted. It was proved that there is an effect from different participant grades in MG Mechanics (H(2) = 7.872, *p* = 0.02 < 0.05) and in MG Aesthetics (H(2) = 8.247, *p* = 0.016 < 0.05), but not in MG Intrinsic Motivation (H(2) = 2.498, *p* = 0.287). As seen in Table 7, the 1st grade leads to higher MGM and MGA.

**Table 7:** Test statistics [Kruskal Wallis Test—Grouping Variable: grade]

Ranks			
	Grade	N	Mean rank
MGM	1st	17	93.94
	2nd	77	66.12
	3rd	43	64.29
	Total	137	
MGA	1st	17	93.59
	2nd	77	64.53
	3rd	43	67.29
	Total	137	

In order to examine possible relation between participants’ gender and the factors, Mann–Whitney Test was conducted because there are two tested group. It was proved that there is no effect from gender in MG Intrinsic Motivation (*p* = 0.153), in MG Aesthetics (*p* = 0.415) or in MG Mechanics (*p* = 0.898).

In order to investigate whether pupils’ opinion on Intrinsic Motivation (IM) role of MG is related to their opinion on Gamification Motivators (GM) of MG, Spearman’s rho test is conducted (Table 8).

**Table 8:** Correlation coefficient between IM and MGM

		IM	MGM
**Spearman's rho**			
IM	Correlation Coefficient	1000	.309**
	Sig. (2-tailed)		< .001
	N	137	137
MGM	Correlation Coefficient	.309**	1000
	Sig. (2-tailed)	< .001	
	N	137	137

**Correlation is significant at the 0.01 level (2-tailed)

It seems that there is a positive correlation between IM and MGM (*p* < 0.05 and rho = 0.31).

In order to investigate whether pupils’ opinion on IM role of MG is related to their opinion on MG Aesthetics, Spearman’s rho test is conducted (Table 9).

**Table 9:** Correlation between IM and MGA

		IM	MGA
**Spearman's rho**			
IM	Correlation Coefficient	1000	.429**
	Sig. (2-tailed)		< .001
	N	137	137
MGA	Correlation Coefficient	.429**	1000
	Sig. (2-tailed)	< .001	
	N	137	137

**Correlation is significant at the 0.01 level (2-tailed)

It seems that there is a positive correlation between IM and MGA (*p* < 0.05 and rho = 0.43).

## Discussion

MG was essentially the tool used to study OLM, OSLM, and gamification motivation techniques and mechanisms that a digital game should have to enhance motivation. Research has demonstrated the contribution of these mechanisms to motivation, and they could constitute recommendations for digital games’ designers. According to relevant bibliography OLMs can motivate users (Brusilovsky et al., 2011; Bull et al., 2007; Bull & Kay, 2007, 2013; Hsiao et al., 2010; Mitrovic & Martin, 2007), while games with OSLM elements have an intense social aspect (social interaction, competition or cooperation) that can increase player involvement (Ling et al., 2005; Sweetser & Wyeth, 2005; Yee, 2006a, 2006b). The existing research concerning the comparison between OLM and OSLM, has pointed out that OSLM outmatches OLM (Bull & Gardner, 2009; Shi et al., 2014; Somyürek et al., 2020). As far as motivation is concerned this research has pointed out something different, as pupils seem to be intrinsically motivated in higher degree when the digital game opens the learner model without the social comparison element, which indicates pupil reluctance to being exposed to their peers. On the other hand, both versions (2 and 3) scored higher in terms of pupil opinion about MG Mechanics and MG Aesthetics compared to the game version without any such features (version 1).

Pupils’ age (as depicted by the grade they attend) seems to play a role in assessing MG Mechanics and MG Aesthetics, as younger pupils (1^st^ grade) due to the more enthusiasm they expressed when participating in the pilot test of MG, had a higher opinion on these 2 factors in comparison to older pupils, while none of the three factors (IM, MGM and MGA) is influenced by the different gender.

The study has indicated that Intrinsic Motivation is positively correlated with MG Mechanics and in this case with Gamification Motivators (due to the fact that the selected questions for the MGM factor also depict the Gamification Motivators factor). This finding is particularly significant as it demonstrates the direct bond that gamification motivators have with intrinsic motivation. Gamification motivators clearly support extrinsic motivation (Amir & Ralph, 2014; Dicheva et al., 2014; Kapp, 2012; Muntean, 2011), but due to the intrinsic psychological needs they support it could be supported that they are also related to intrinsic motivation (Matallaoui et al., 2017; Richter et al., 2015a, 2015b; Yang et al., 2020), an observation supported by this study, too. Furthermore, Intrinsic Motivation has proved to positively relate to MG Aesthetics, which is in agreement with relative research (Garcìa-Vergara et al., 2015; Alexiou & Schippers, 2018) as Aesthetics represent not only what a player can sense (see/hear), but also the player’s feelings while interacting with the game.

As teachers play a very significant role in adopting (or not) digital games in the teaching process (Baek, 2008; Bakar et al., 2006; Bourgonjon et al., 2013; De Grove et al., 2012; Leonardou, Rigou, Panagiotarou, et al., 2021a, 2021b; Teo, 2008), it is very important to record their experience about using MG themselves and their pupils too. Teachers’ evaluation of MG has been quite encouraging, as they seem to be convinced of the entertaining, educational and assessing role MG can play in formal instructional conditions which is also indicated by relative work about MG (Leonardou et al., 2021a, 2021b) and about digital game in general (Allsop et al., 2013; Barbour et al., 2009; Bourgonjon et al., 2013; Clark & Mayer, 2016; De Grove et al., 2012; Huizenga et al., 2017; Proctor & Marks, 2013; Ruggiero, 2013).

In addition to the aforementioned survey conducted with teachers in Greek public schools, the teachers that were involved in the current study were also interviewed. A semi-structured interview took place with the 8 teachers (2 male, 6 female with an average age of 47,6 years) that participated with their pupils in the pilot use of MG. Teachers considered MG as a useful tool in the educator’s hands: enjoyable, very interesting and original. MG was also characterized as being very creative and easily accessible by children. Pupils can practice propaedia in a pleasant way and gradually improve their skills. Regarding the idea of using MG as part of the teaching procedure, either as homework or as part of school practice, all teachers believe that MG can be a part of their teaching procedure especially as homework, without underestimating the educational role it can play in the classroom as well. Teachers stated that pupils certainly benefited in learning propaedia by using the game (compared to traditional methods), and this especially applies for low-achievers or pupils with daily game practice. 50% of teachers didn’t use MG to watch their pupils’ performance. 5 of the teachers used MG as a way of practicing propaedia, while only one teacher used it after the end of the (traditional) instructional procedure, as an activity of relaxation and learning. 3 teachers used MG as a tool of pupil assessment, which is expected taking into account teachers’ concerns about parental intervention in pupils playing at home, as scores in MG sometimes were higher than pupil’s competencies. The absence of high rates in pupils’ engagement can be attributed—according to teachers- to the fact that parents in many cases didn’t allow their kids to use devices in an effort to balance their long hours on computer screens due to intensive distance learning imposed in Greece during the pandemic. A suggestion for MG improvement was to include numbers for multiplication tables of 11, 12, 13 for high achievers. Another suggestion was to include visual explanations as feedback for common mistakes in propaedia, an approach that could increase learning in the game.

The fact that the MG pilot use took place during the COVID-19 pandemic, a period that primary schools in Greece operated in distance-learning conditions, led to fewer teachers willing to participate with their own pupils in the testing of the game. This fact, combined with parents discouraging their kids to spend more time on a computer screen narrowed the number of participating pupils, as well as the frequency of their game sessions, which in turn did not allow us to collect enough data on pupil multiplication skills enhancement and analyze the learning effectiveness of the 3 different game versions. Given this limitation, a future MG study should take place during the schoolyear under normal circumstances, while pupils can practise multiplication with MG being used primarily in the classroom (computer laboratory), under the teacher’s guidance and control. Furthermore, pre-tests and post-test will show how MG versions influence pupils’ learning in comparison to a control group that won’t use MG.

## Conclusion

The primary question of this study is whether OLM and OSLM mechanisms, when used in a digital game, offer higher motivation. Furthermore, the study investigates whether MG aesthetics and mechanics support players’ intrinsic motivation. Both objectives were investigated and supported by the design, implementation and pilot use of the Multiplication Game (MG). MG is a digital learning activity that supports pupils in achieving multiplication competence and provides teacher a dashboard to assess and watch own pupils’ performance. The game enriched with gamification elements to engage and motivate participants. Thus, MG can be considered as a bridge between the formal teaching procedure and digital games, offering pupils the opportunity to exercise multiplication tables through a computer game, while teachers can track own pupils’ performance. MG opens the pupil learner model through suitable visualizations to both pupils and teachers, to support metacognitive skills and pupils’ engagement. Moreover, gamification motivators have been incorporated in MG’s last version to offer pupils higher motivation. According to the data analysis pupils’ opinion about Aesthetics and Mechanics of MG is higher when MG offers OLM characteristics, and pupils are more motivated in this case. Gender seems to play no role in accessing MG Aesthetics, Mechanics or Intrinsic Motivation. Intrinsic Motivation seems to be positively correlated with MG Aesthetics, MG Mechanics and Gamification Motivators. The last finding is very important as Gamification Motivators have basically a bond with extrinsic motivation, but through this research another bond, that of intrinsic motivation, was highlighted.

Both pupils and teachers seem to have enjoyed using MG and believe in its usefulness and potential. It is among future goals to incorporate visual explanations as feedback for common misconceptions in multiplication tables. It remains an open question to investigate whether the 3 versions of the game contribute and to what extent to the acceleration of learning and whether elements that enhance competition among children (OSLM) are effective motivation tools.

## Data Availability

The datasets generated during and/or analysed during the current study are available from the corresponding author on reasonable request.

## Appendix -Questionnaire

**Table:**

IM1	I want to play the game because I enjoy learning multiplication facts
IM2	I want to play the game because I want to succeed in learning multiplication facts
IM3	I want to play the game because it is entertaining
MGM1	I liked selecting my avatar
MGM2	I liked that the panda and the cat gave me information in the game
MGM3	I liked winning coins when giving a correct answer
MGM4	I liked watching my performance after completing each level and when completing the game
MGM5	I liked watching my performance in comparison to my previous plays
MGM6	I liked watching my performance in comparison to the total performance of my classmates
MGA1	I felt happy while I was playing the game
MGA2	I enjoyed the sounds in the game
MGA3	I enjoyed the colors and graphics in the game
